# Calcitonin gene-related peptide receptor antagonist ubrogepant for the treatment of acute migraine: A meta-analysis

**DOI:** 10.1097/MD.0000000000024741

**Published:** 2021-02-26

**Authors:** Zizhen Zhang, Yunfeng Shu, Yun Diao, Yang Du, Lizhi Chen, Ying Liu, Biao Du

**Affiliations:** a School of Pharmacy, Southwest Medical University, Luzhou Sichuan; b School of Pharmacy, North Sichuan Medical College, Nanchong Sichuan; c Department of Psychosomatic Medicine; d Department of Pharmacy, the Affiliated Three Gorges Hospital of Chongqing University, Chongqing, China.

**Keywords:** acute migraine, calcitonin gene-related peptide receptor antagonist, meta-analysis, psychotherapy, ubrogepant

## Abstract

**Background:**

The objective of this study is to systematically evaluate the efficacy and safety of the calcitonin gene-related peptide (CGRP) receptor antagonist ubrogepant for the treatment of acute migraine.

**Methods:**

Randomized controlled trials (RCTs) of ubrogepant for treatment of acute migraine were identified in PubMed, MEDLINE, EMBASE, and the Cochrane Library from database establishment to June 2020; we also searched ClinicalTrials.gov manually during the same period. Then, RevMan 5.3 software was used to perform a meta-analysis on each outcome measure.

**Results:**

A total of 5 RCTs involving 4903 patients were included; there were 3358 cases in the ubrogepant group and 1545 cases in the placebo group. The meta-analysis showed the following results: at 2 hours postdose, the percentages of participants reporting pain relief and the absence of photophobia, nausea, and phonophobia were significantly higher in the ubrogepant group than in the placebo group (odds ratio [OR] = 1.71, 95%CI: 1.48–1.97, *P* < .00001; OR = 1.33, 95%CI: 1.22–1.45, *P* < .00001; OR = 1.07, 95%CI: 1.03–1.11, *P* = .0006; OR = 1.21, 95%CI: 1.14–1.28, *P* < .00001). The incidence of common adverse events was similar between the 2 groups (*P* > .05).

**Conclusion:**

Ubrogepant is effective and safe for the treatment of acute migraine.

**Registration number:**

PROSPERO CRD42019145286.

## 1 Introduction

Migraine is one of the most common nervous system diseases. It is characterized by recurrent unilateral pulsatile headache, with sensitivity to movement, visual stimulation, sound, and other sensory stimuli.^[1]^ Most migraines cause discomfort for hours or days after the attack and are often accompanied by fatigue and other sequelae.^[2]^ Migraines can occur at any time and commonly occur during sleep, upon awakening, or shortly after rising in the morning,^[3,4]^ which is very inconvenient for patients.

About 1 billion people worldwide are affected by migraine, the ratio of female to male is 3:1 and the high incidence of migraine is between 35 and 39 years old.^[5,6]^ In addition to adults, recurrent headache occurs in one-third to half of children and adolescents.^[7]^

The 5-hydroxytryptamine receptor agonist class triptans, discovered in the early 1990s, constitutes the only class of specific drugs developed and approved for the treatment of acute migraine in the past 20 years, and treatment with triptans is the standard protocol recommended by various guidelines.^[8]^ However, triptans have adverse effects, and the use of triptans significantly increases the risk of cardiovascular and cerebrovascular events.^[9,10]^ Calcitonin gene-related peptide (CGRP) can dilate the cerebral arteries and mediate neurogenic inflammation of the dura, which plays a key role in the pathophysiological mechanism underlying migraine.^[11]^ As an oral CGRP receptor antagonist, ubrogepant mainly acts on the smooth muscle cells of the microvascular wall to control peripheral vascular resistance.^[12,13]^ Ubrogepant may be able to meet the acute treatment needs of patients with migraine who are intolerant or unresponsive to triptans.^[14]^ This study systematically evaluated the efficacy and safety of ubrogepant for the treatment of acute migraine to provide evidence that can serve as a reference in the subsequent clinical application of the drug.

## 2 Materials and methods

### 2.1 Inclusion and exclusion criteria

#### 2.1.1 Type of study

Randomized controlled trial (RCT).

#### 2.1.2 Type of subjects

(1) Patients had at least a 1-year history of migraine with or without aura as defined by the International Headache Society (IHS) criteria 1.1 and/or 1.2.
(2) Patients were 18 years old or older (sex and region were not considered).
(3) Patients had moderate or severe migraine attack 2 to 8 times per month.

#### 2.1.3 Intervention measures

(1) Experimental group: single drug treatment with ubrogepant, divided into subgroups according to dose.
(2) Placebo group: placebo single-drug control.

#### 2.1.4 Outcome measures

The primary outcome measure was the percentage of subjects experiencing pain relief at 2 hours postdose. The secondary outcome measures were as follows: the percentage of subjects without photophobia at 2 hours postdose; the percentage of subjects without nausea at 2 hours postdose; and the percentage of subjects without phonophobia at 2 hours postdose. The safety outcome measure was the incidence of common adverse effects.

#### 2.1.5 Study exclusion criteria

(1) Multiple published studies with the same data;
(2) reviews, retrospective studies, pharmacokinetics studies, etc;
(3) cohort studies; and
(4) open clinical trials without placebo control.

### 2.2 Search strategy

PubMed, MEDLINE, EMBASE, the Cochrane Library, and other databases were searched for clinical RCTs on acute migraine treated with ubrogepant from the establishment of the database to June 2020. The search terms used were “ubrogepant,” “MK-1602,” “migraine,” “Calcitonin gene-related peptide,” “CGRP,” “Calcitonin gene-related peptide receptor antagonist,” “CGRP receptor antagonist,” “randomized controlled trial,” “RCT,” and “controlled clinical trial.”

### 2.3 Literature screening and data extraction

Endnote X7 software was used to remove duplicates in the included literature. Two researchers read the titles, abstracts, and full texts independently, screened the RCTs and determined whether they met the standards. If there was any disagreement, a third researcher was consulted. The extracted data included the basic information of the included study, the data pertaining to the outcome measures and the quality indicators of the included studies. Then, the 2 researchers cross-checked the above information.

The risk of bias was evaluated by the Cochrane system evaluator in Handbook 5.1.0,^[15]^ which evaluates

(1) random sequence generation;
(2) the hidden allocation scheme;
(3) the blinding method;
(4) the handling of incomplete data;
(5) the selective reporting of results;
(6) other biases.

RevMan 5.3 software provided by the Cochrane Collaboration Network was used for the statistical analysis. First, *χ*^2^ tests were used to assess heterogeneity, and the test level was α = 0.1. When there was no statistical heterogeneity among the studies (*P* > .1, *I*^2^ ≤ 50%), the fixed effect model was used for the meta-analysis. If statistical heterogeneity was found among the studies (*P* < .1, *I*^2^ > 50%), a random effect model was used, and subgroup and sensitivity analyses were carried out if necessary. For continuous data, the effect index was mean difference (MD) and its confidence interval (95%CI); for binary data, the effect index was relative risk (RR) or odds ratio (OR) and its 95%CI.

## 3 Results

### 3.1 Literature retrieval and basic information

**Figure 1:**
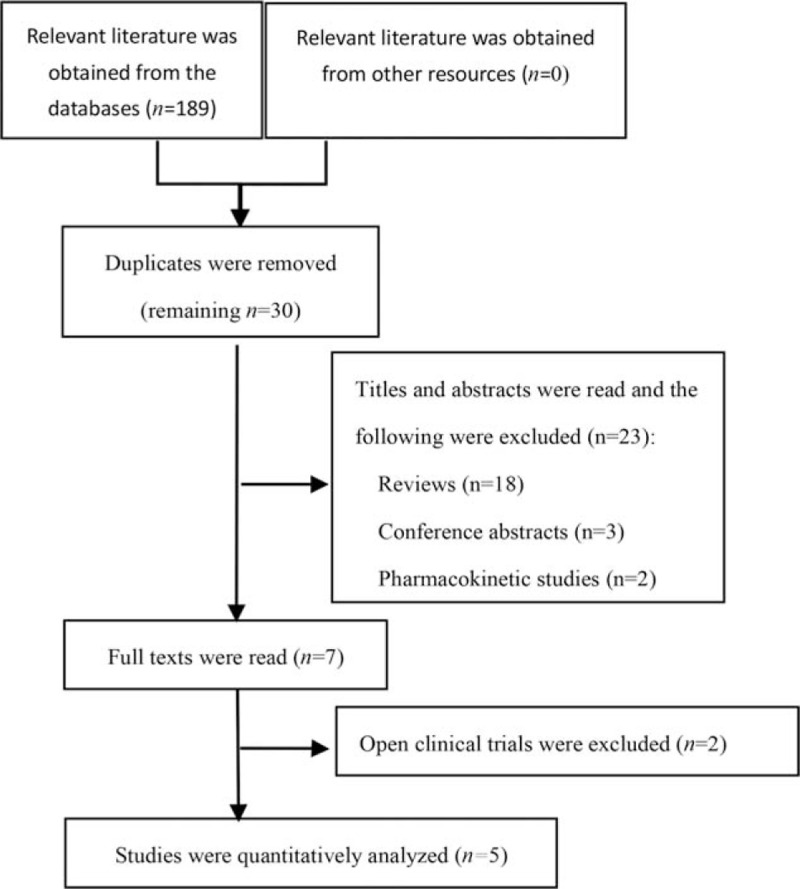
Study retrieval flow chart.

A total of 189 articles were obtained. After screening, 5 studies were included in the quantitative analysis, with a total of 4903 patients. Ethical approval was not necessary, as all the included papers have passed the ethical review. There were 3358 cases in the ubrogepant group and 1545 cases in the placebo group. The flow chart of literature retrieval is shown in Figure 1, and the basic information of the literature is shown in Table 1.

**Table 1:** Basic information of the included literature.

Study	Year	Group (dose, mg)	No.	Female/Male	Age, yr	Outcome measures
01657370^[13]^	2012	Ub = 1	28	26/2	NA	①②③④⑤
		Ub = 10	26	22/4		
		Ub = 25	28	23/5		
		Ub = 50	28	26/2		
		Ub = 100	27	18/9		
		Placebo	28	25/3		
voss2016^[14]^	2012	Ub = 1	138	95/12	39.6 ± 10.7	①②③④⑤
		Ub = 10	139	92/16	41.1 ± 10.9	
		Ub = 25	139	91/13	41.4 ± 11.5	
		Ub = 50	139	92/14	40.7 ± 12.3	
		Ub = 100	139	90/12	41.9 ± 11.0	
		Placebo	139	99/14	40.8 ± 11.4	
Lipton2019^[15]^	2018	Ub = 25	561	501/60	41.6 ± 12.3	①②③④⑤
		Ub = 50	562	497/65	41.0 ± 12.4	
		Placebo	563	494/69	41.5 ± 12.2	
Dodick2019^[16]^	2017	Ub = 50	556	493/63	40.2 ± 12.0	①②③④⑤
		Ub = 100	557	479/78	40.7 ± 12.4	
		Placebo	559	491/68	40.5 ± 12.2	
Goadsby2019^[17]^	2018	Ub = 100	260	140/116	NA	①②③④⑤
		Placebo	256	141/119		

NA = not available, Ub = ubrogepant; ① percentage of participants reporting pain relief at 2 h postdose; ② percentage of participant reporting absence of photophobia at 2 h postdose; ③ percentage of participants reporting absence of nausea at 2 h postdose; ④ percentage of participants reporting absence of phonophobia at 2 h postdose; ⑤ common adverse effects.

### 3.2 Basic characteristics of the included studies and bias risk assessment results

**Figure 2:**
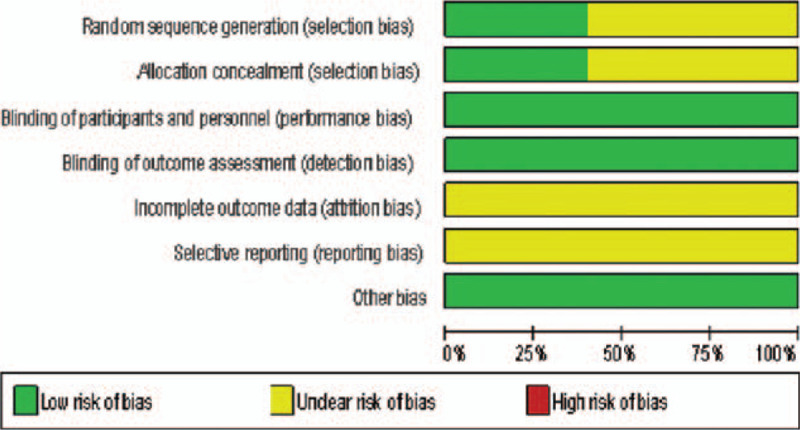
Risk of bias assessment of included studies.

**Figure 3:**
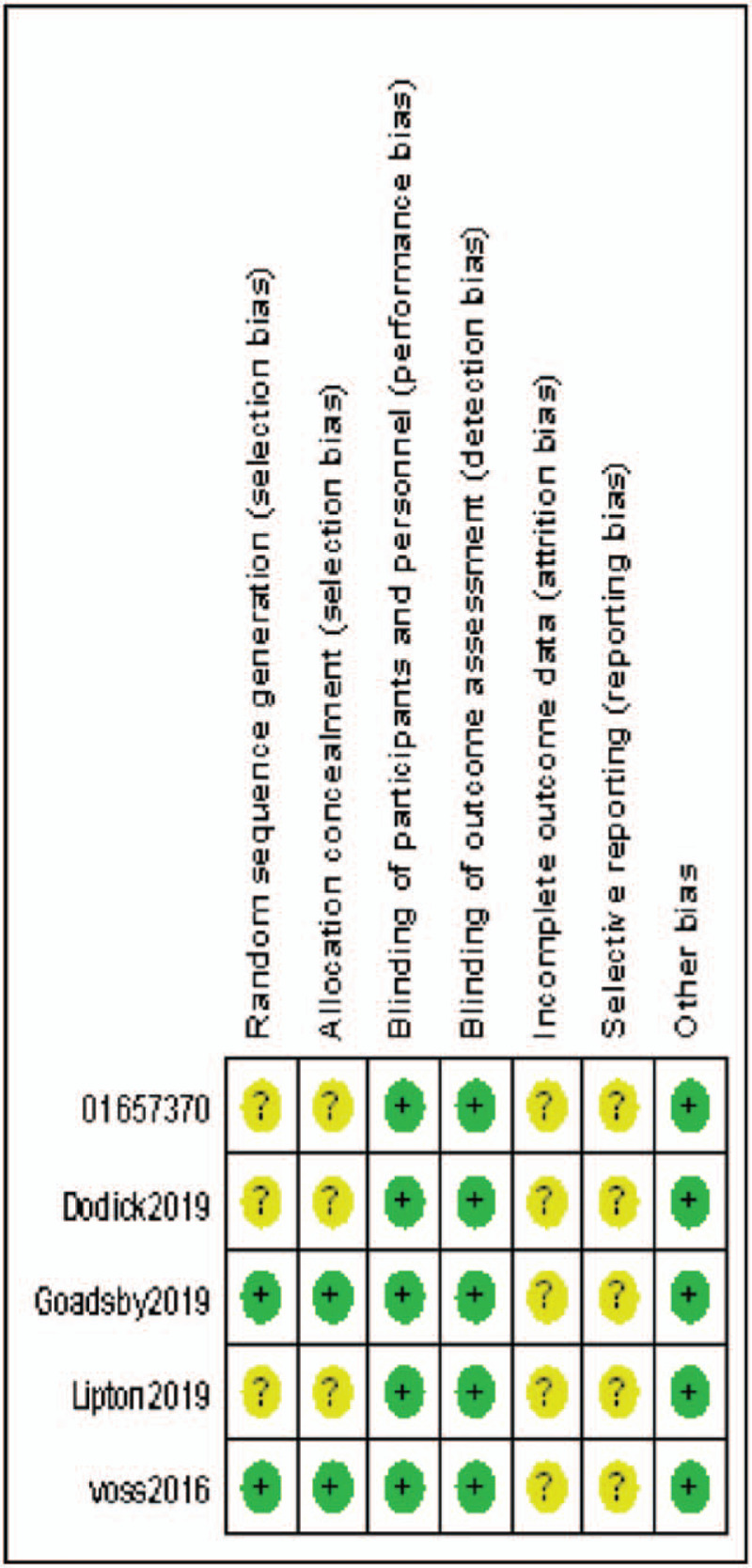
Risk of bias assessment of included studies.

The 5 included studies^[16–20]^ were all in English and were randomized, double-blind RCTs. The follow-up time and outcome measures were generally comparable, and the samples were representative. Two papers^[17,20]^ reported specific random sequence generation methods, while others only mentioned random grouping but did not report the specific methods. All studies^[16–20]^ reported the specific numbers of missed visits and dropouts. The quality of the included studies is shown in Table 2, and the assessment of the risk of bias is shown in Figures 2 and 3.

**Table 2:** Quality characteristics of the included literature.

Study	Random sequence generation	Hidden allocation scheme	Blinding method	Incomplete results	Selective reporting of results	Other biases
01657370^[13]^	Unclear	Unclear	Double blind	Low risk	Low risk	Low risk
Voss2016^[14]^	Cross voice response system	Low risk	Double blind	Low risk	Low risk	Low risk
Lipton2019^[15]^	Unclear	Low risk	Double blind	Low risk	Low risk	Low risk
Dodick2019^[16]^	Unclear	Low risk	Double blind	Low risk	Low risk	Low risk
Goadsby2019^[17]^	Computer generated randomization scheme	Low risk	Double blind	Low risk	Low risk	Low risk

### 3.3 Meta-analysis results

**Figure 4:**
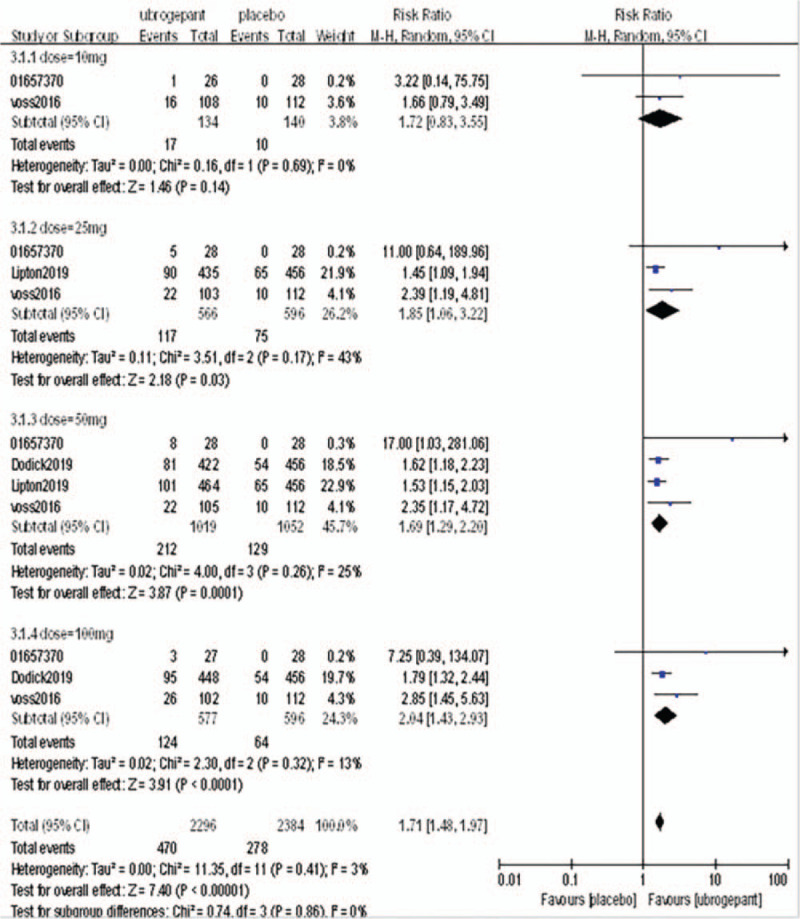
Comparison of the percentage of subjects with pain relief within 2 h after the first administration between the experimental group and the placebo group.

Primary outcome measures: The primary outcome measure was the percentage of subjects with pain relief at 2 hours postdose. Four RCTs^[16–19]^ involving 4406 patients were included. Meta-analysis with a random effect model showed a significant difference between the experimental group and the placebo group (OR = 1.71, 95%CI: 1.48–1.97, *P* < .00001), subgroup analysis showed that there was no significant difference between the dose of 10 mg and 25 mg (*P* = .14, *P* = .03) compared with placebo group. When the dose was increased to 50 mg and 100 mg, the efficacy of the experimental group was significantly better than that of the placebo group (*P* = .0001, *P* < .0001) as shown in Figure 4.

Secondary outcome measures: The secondary outcome measures were the percentages of subjects without photophobia, nausea, and phonophobia at 2 hours postdose.

**Figure 5:**
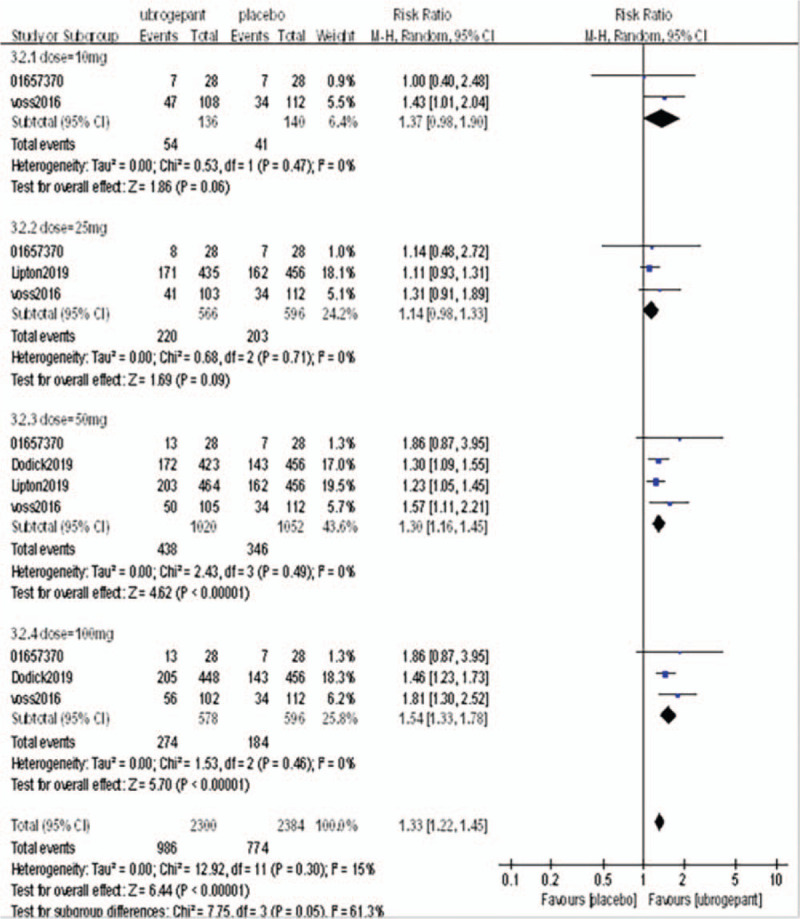
Comparison of the percentage of subjects with photophobia 2 h after initial administration between the experimental group and the placebo group.

**Figure 6:**
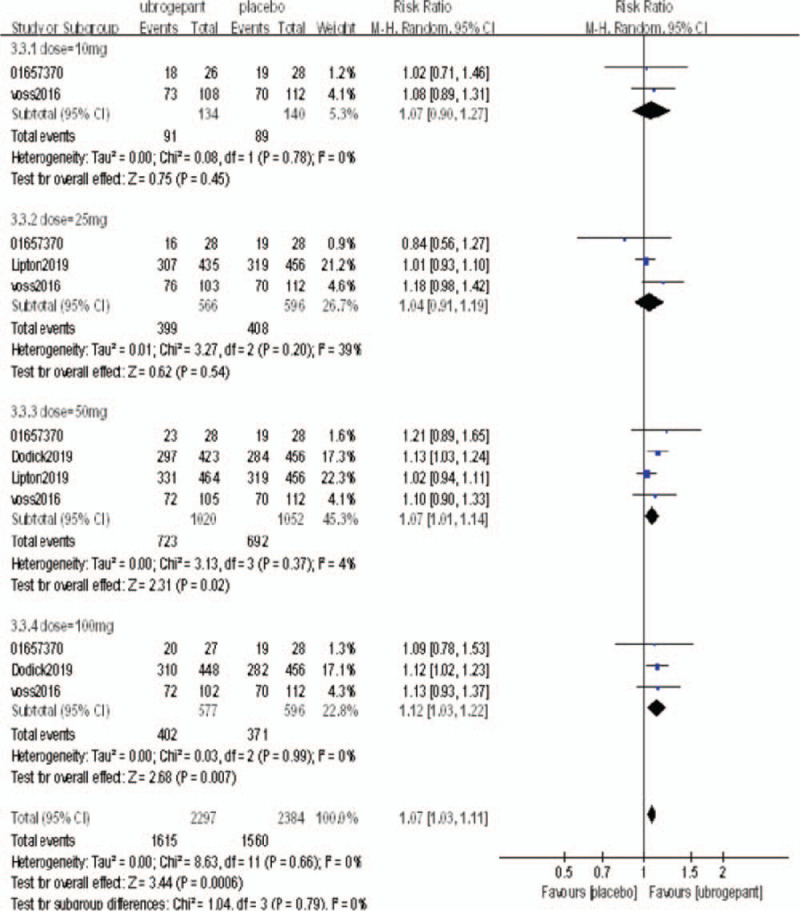
Comparison of the percentage of subjects with no nausea 2 h after the initial administration between the experimental group and the placebo group.

**Figure 7:**
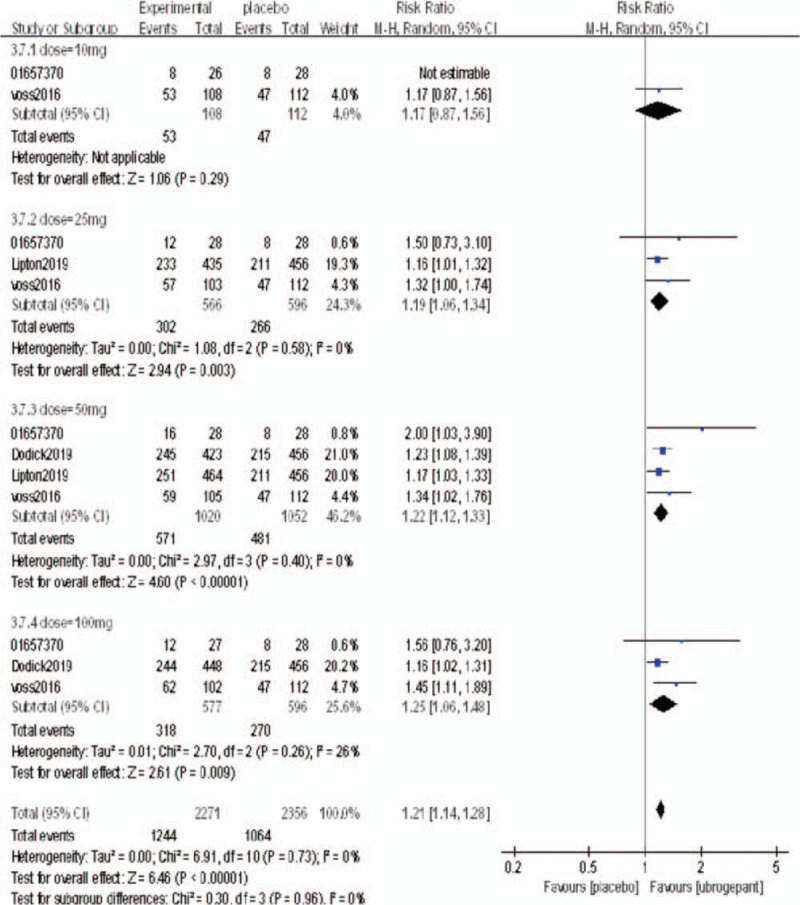
Comparison of the percentage of subjects without phonophobia 2 h after initial administration between the experimental group and the placebo group.

A total of 4 RCTs^[16–19]^ reported the percentages of subjects without photophobia, nausea, and phonophobia at 2 hours postdose. The above outcome measures were analyzed with a random effect model. After a single administration, the secondary outcome measures in the experimental group were better than those of the placebo group, and the differences were statistically significant (RR = 1.33, 95%CI = 1.22–1.45, *P* < .00001; RR = 1.07, 95%CI = 1.03–1.11, *P* = .0006; RR = 1.21, 95%CI = 1.14–1.28, *P* < .00001, respectively). The above 3 secondary outcome measures were divided into subgroups according to the dose. In the without photophobia outcome measure, there was no significant difference between the 2 groups (*P* = .06, *P* = .09) in the dose of 25 mg and 50 mg, but there was significant difference in the dose of 50 mg and 100 mg (*P* < .00001, *P* < .00001). In the without nausea outcome measure, the difference was not statistically significant when the dose was 10 mg, 25 mg, 50 mg (*P* = .45, *P* = .54, *P* = .02), and the difference was statistically significant when the dose was 100 mg (*P* = .07). In the phonophobia outcome measure, there was no significant difference when the dose was 25 mg (*P* = .06), but there was significant difference when the dose was 25 mg, 50 mg, 100 mg (*P* < .003, *P* < .00001, *P* < .00001). The results of the meta-analysis are shown in Figures 5–7.

### 3.4 Meta-analysis of safety indicators

The incidence of common adverse reactions was reported in 3 studies,^[16,17,20]^ including headache (31/393 vs 25/288), oropharyngia (36/392 vs 10/288), nasopharynx (18/393 vs 18/288), nausea (49/920 vs 16/401), dizziness (39/920 vs 9/401), diarrhea (11/393 vs 8/288), fatigue (8/393 vs 7/288) had no significant difference compared with placebo group (*P* > .05) (Table 3).

**Table 3:** Meta-analysis results of comparison of common adverse effects between the 2 groups.

		Incidence, %		Effect estimates			
Outcome measures	Model	Ubrogepant	Placebo	RR	95%CI	*P*	*I*^2^ (%)
Headache	Random	7.89%	8.68%	1.17	0.71–1.93	.53	0
Oropharyngeal pain	Random	9.18%	3.47%	2.10	0.99–4.48	.30	42
Nasopharyngitis	Random	4.58%	6.25%	0.83	0.43–1.60	.59	0
Nausea	Random	5.33%	3.99%	1.24	0.67–2.29	.55	0
Dizziness	Random	4.24%	2.24%	1.38	0.25–7.70	.71	78
Diarrhea	Random	2.80%	2.78%	1.06	0.42–2.65	.91	0
Fatigue	Random	2.04%	2.43%	0.88	0.32–2.40	.77	0

95%CI = confidence interval, RR = relative risk.

### 3.5 Sensitivity analysis of each index

Sensitivity analyses were carried out on the indexes of effectiveness and safety. After changing the effect model (fixed or random) and removing the maximum or minimum weight proportion, the results of the meta-analysis were not significantly different from those of the original analysis, indicating low sensitivity and high stability of the research results (Table 4).

**Table 4:** Sensitivity analysis of effectiveness indicators.

	Fixed effect model			Excluded the RCT with maximum weight			Excluded the RCT with minimum weight		
Outcome measures	RR	95%CI	*P*	RR	95%CI	*P*	RR	95%CI	*P*
Pain relief	1.75	1.52–2.00	<.00001	1.78	1.50–2.11	<.00001	1.70	1.47–1.96	<.00001
Absence of photophobia	1.32	1.22–1.42	<.00001	1.36	1.23–1.51	<.00001	1.34	1.22–1.47	<.00001
Absence of nausea	1.07	1.03–1.12	.0004	1.09	1.04–1.14	.002	1.07	1.03–1.12	.0004
Absence of phonophobia	1.21	1.14–1.28	<.00001	1.20	1.13–1.29	<.00001	1.21	1.14–1.28	<.00001

95%CI = confidence interval, RCTs = randomized controlled trials, RR = relative risk.

## 4 Discussion

A total of 5 RCTs were included in this study. The baseline and outcome data were relatively complete and balanced, with high comparability and quality of the included literature. The aim of this study was to evaluate the efficacy and safety of ubrogepant for the treatment of acute migraine. The analysis results of 5 RCTs showed that the percentages of subjects with pain relief and the absence of photophobia, nausea, and phonophobia at 2 hours postdose were significantly higher in the experimental group than in the placebo group (*P* < .05). However, the effect of different doses of ubrogepant was not stable. Subgroup analysis by dose showed that when the dose was 10 mg and 25 mg, there were no significant differences in the 4 effective outcome measures between the experimental group and the placebo group. When the dose was increased to between 50 mg and 100 mg, the outcome measures in the experimental group were significantly better than those in the placebo group, which is consistent with the research results obtained by Do's group.^[21]^ It is recommended that the starting dose of ubrogepant should be at least 25 mg, and the analgesic effect is enhanced as the dose increases.

We analyzed the safety of the included studies, that is, the incidence of common adverse reactions and serious adverse reactions. The common adverse effects, such as headache, oropharyngeal pain, nasopharyngitis, nausea, dizziness, diarrhea and fatigue, in the experimental group were similar to those in the placebo group, and the incidence rates were low, suggesting that patients tolerated ubrogepant treatment well. As severe adverse effects, Lipton^[18]^ reported 1 patient in the experimental group had severe adverse effects on the nervous and urinary systems, while the placebo group had no severe adverse effects; Voss^[17]^ reported that 1 patient in the experimental group had severe adverse effects (myoclonus), while the placebo group had no severe adverse effects; Goasby^[20]^ reported that 2 patients in the experimental group had severe adverse effects (1 subject had a selective abortion, and the other subject had abdominal pain, arthralgia, back pain, musculoskeletal pain, and neck pain related to a motor vehicle accident that occurred on day 55), while one severe adverse effect (selective abortion) occurred in the placebo group, but the above severe adverse effects were not considered to be directly related to the interventions. The results of a long-term open clinical trial^[22]^ showed that the incidence of severe adverse effects in the experimental group (2.58%) was lower than that in the conventional treatment group (4.08%). There was no significant difference between the incidence of common adverse effects in the experimental group (32.35%) and the conventional treatment group (31.65%). This conclusion is similar to the research results in this paper, indicating that the safety and tolerability of ubrogepant are good.

However, this study also has limitations:

(1) Due to the limitation of the number and language of the included literature, the sample size is small, which may have led to publication bias, affecting the reliability of the results.
(2) One of the RCTs in this study had no relevant published literature and lacked descriptions of the randomization scheme and allocation concealment method. Thus, there may have been implementation bias or other bias, which could reduce the reliability of the results.
(3) Due to the low incidence of adverse effects, we did not conduct a subgroup analysis of adverse effects. It is unknown whether an increase in the dose of ubrogepant increases the incidence of common adverse effects.
(4) None of the participants in the studies had cardiovascular or cerebrovascular diseases; therefore, the safety of ubrogepant in this group of people cannot be determined.
(5) The intervention measures in 5 studies were the single administration of ubrogepant, and the follow-up time was short; therefore, it was difficult to evaluate the long-term efficacy and safety of ubrogepant objectively. The results of this study should serve as a reference only. To obtain more stable results, more high-quality studies are needed.

## Author contributions

**Conceptualization:** Zhizhen Zhang.

**Data curation:** Yunfeng Shu.

**Formal analysis:** Yunfeng Shu.

**Funding acquisition:** Biao Du.

**Investigation:** Yun Diao.

**Methodology:** Yun Diao.

**Project administration:** Yang Du.

**Resources:** Yang Du.

**Software:** Lizhi Chen.

**Supervision:** Zhizhen Zhang.

**Validation:** Lizhi Chen.

**Visualization:** Ying Liu.

**Writing – original draft:** Zhizhen Zhang.

**Writing – review & editing:** Ying Liu.
